# Use of Ultrathin Bronchoscope on a Need Basis Improves Diagnostic Yield of Difficult-to-Approach Pulmonary Lesions

**DOI:** 10.3389/fmed.2020.588048

**Published:** 2020-12-15

**Authors:** Yoichi Nishii, Yuki Nakamura, Kentaro Fujiwara, Kentaro Ito, Tadashi Sakaguchi, Yuta Suzuki, Kazuki Furuhashi, Tetsu Kobayashi, Taro Yasuma, Corina N. D'Alessandro-Gabazza, Esteban C. Gabazza, Fumihiro Asano, Osamu Taguchi, Osamu Hataji

**Affiliations:** ^1^Respiratory Center, Matsusaka Municipal Hospital, Matsusaka, Japan; ^2^Department of Pulmonary and Critical Care Medicine, Mie University Faculty and Graduate School of Medicine, Tsu, Japan; ^3^Department of Immunology, Mie University Faculty and Graduate School of Medicine, Tsu, Japan; ^4^Gifu Prefectural General Medical Center, Gifu, Japan

**Keywords:** ultrathin bronchoscope, peripheral pulmonary lesion, diagnostic bronchoscopy, endobronchial ultrasound, thin bronchoscope

## Abstract

There are cases of peripheral lung nodules that are difficult to approach despite using ancillary diagnostic devices during multimodal bronchoscopy. The use of ultrathin bronchoscopes has shown superiority over standard thin bronchoscopes. We retrospectively evaluated whether substitution of the thin-bronchoscope by the ultrathin device during multimodal bronchoscopy improves lesion ultrasound visualization and diagnostic yield in patients with difficult-to-approach pulmonary lesions. The study comprised 44 out of 338 patients that underwent multimodal bronchoscopy at Matsusaka Municipal Hospital. The thin-bronchoscope with an external diameter of 4 mm was substituted by the ultrathin-bronchoscope with an external diameter of 3 mm when the radial endobronchial ultrasound showed that the probe position was not within the target lesion. The median diameter of the pulmonary tumors was 17.5 mm (range: 6.0–5.2.0 mm). The endobronchial ultrasound showed the probe's position adjacent to the lesion in 12 cases and no visible lesion in 32 cases using a thin-bronchoscope. However, the endobronchial ultrasound views changed from adjacent to the lesion to within the lesion in nine cases, from no visible lesion to within the lesion in 17 cases, and from no visible lesion to adjacent to the lesion in nine cases after bronchoscope substitution. After substitution, the diagnostic yield was 80.8% in cases with the radial probe within the target lesion, 72.7% in cases with the probe adjacent to the target lesion, and 0% in cases with no visible lesion. The overall diagnostic yield was 65.9% after bronchoscope substitution. The substitution of the thin bronchoscope by the ultrathin device on a need basis improves the position of the radial endobronchial ultrasound probe and diagnostic yield of pulmonary lesions during multimodal diagnostic bronchoscopy.

## Introduction

The development of ancillary techniques for standard bronchoscopy procedures, including virtual bronchoscopy navigation (VBN) and radial-probe endo-bronchial ultrasound (EBUS), has markedly improved the diagnostic yield of peripheral pulmonary lesions of <2 cm (1). The combined use of VBN and radial-probe EBUS increases the diagnostic yield of peripheral lesions to more than 80% (1). The American College of Chest Physicians recommends using the radial-probe EBUS based on studies reporting diagnostic yields of more than 70% (2). The radial EBUS probe positioning within the lesion appears to be a critical factor for obtaining diagnostic material during bronchoscopic procedures (3–5). A previous study reported diagnostic yields of 83% in cases in which biopsy was performed with the radial probe within the target lesion, 61% when the radial probe was located adjacent to the lesion, and only 4% in cases in which the radial EBUS probe was outside the lesion (4). These observations suggest the need to use different technical modality to ensure that the radial EBUS probe's position is within the target lesion during bronchoscopy diagnostic procedures.

The 3-mm ultrathin bronchoscope has several advantages over the standard 4-mm thin bronchoscope (6–9). A previous randomized study has shown convincing evidence that using an ultrathin bronchoscope instead of the thin bronchoscope combined with radial probe EBUS significantly facilitates the accessibility to and selectivity of distal bronchial branches and increases the diagnostic yield of diagnostic bronchoscopy (10). In the present study, we hypothesized that the substitution of the standard thin bronchoscope by the ultrathin device would improve EBUS visualization and diagnostic outcome of pulmonary lesions during multimodal bronchoscopy. To demonstrate this hypothesis, we evaluated the changes in EBUS view and in diagnostic yield after substitution of the thin device by the ultrathin bronchoscope in cases in which the radial EBUS probe's position is not within the lesion during standard bronchoscopy procedures.

## Patients and Methods

### Data Source and Study Design

The study included patients that consulted the Matsusaka Municipal Hospital from March 3, 2018 to May 5, 2019. We reviewed retrospectively the records of 44 patients with pulmonary lesions that underwent multimodal diagnostic bronchoscopy using both thin and ultrathin bronchoscopes. The inclusion criteria were cases in which ultrathin bronchoscope was used as a substitute on a need basis when the EBUS view showed that the radial probe was not within the target lesion during multimodal bronchoscopy using a standard thin bronchoscope. The exclusion criteria consisted of cases in which the purpose of bronchoscopy was re-biopsy, the CT scan showed ground-glass opacity or semi-solid tumors, and/or the target lesion was directly visualized through bronchoscopy. We assessed the following parameters: sex, age, tumor size, echographic and chest X-ray findings, the feasibility of fluoroscopy, presence or absence of bronchus-sign on CT scan, presence of pulmonary lesion at 2.5 cm from the lung hilum and the parietal pleura, availability of pathological lung tissue specimens, and final diagnosis.

### Ethical Statement

The Ethics Committee for Clinical Investigation of the Matsusaka Municipal Hospital approved the study protocol (Approval No J-58-191101-8-7), and the study was performed following the Principles of the Helsinki Declaration. Informed consent was obtained from all patients before the diagnostic procedures.

### Procedures

All patients underwent a lung CT scan (TSX-101AQA, Canon Medical Corporation, Tochigi, Otawara, Japan), and the VBN (DirectPath, Cybernet Systems, Tokyo, Japan) was prepared using the CT DICOM data. We used the thin bronchoscope type P290 (outer diameter, 4.0 mm; working channel diameter, 2.0 mm), the thin bronchoscope type P260F (outer diameter, 4.0 mm; working channel, 2.0 mm), and the ultrathin bronchoscope type MP290F (outer diameter, 3.0 mm; working channel diameter 1.7 mm). All bronchoscopes were from the Olympus Medical Systems, Tokyo, Japan. Before the bronchoscopy procedure, we anesthetized the pharyngeal area by spraying lidocaine (Spray Catheter, Olympus, Tokyo, Japan) and sedated the patients with midazolam and fentanyl (11).

**Figure 1:**
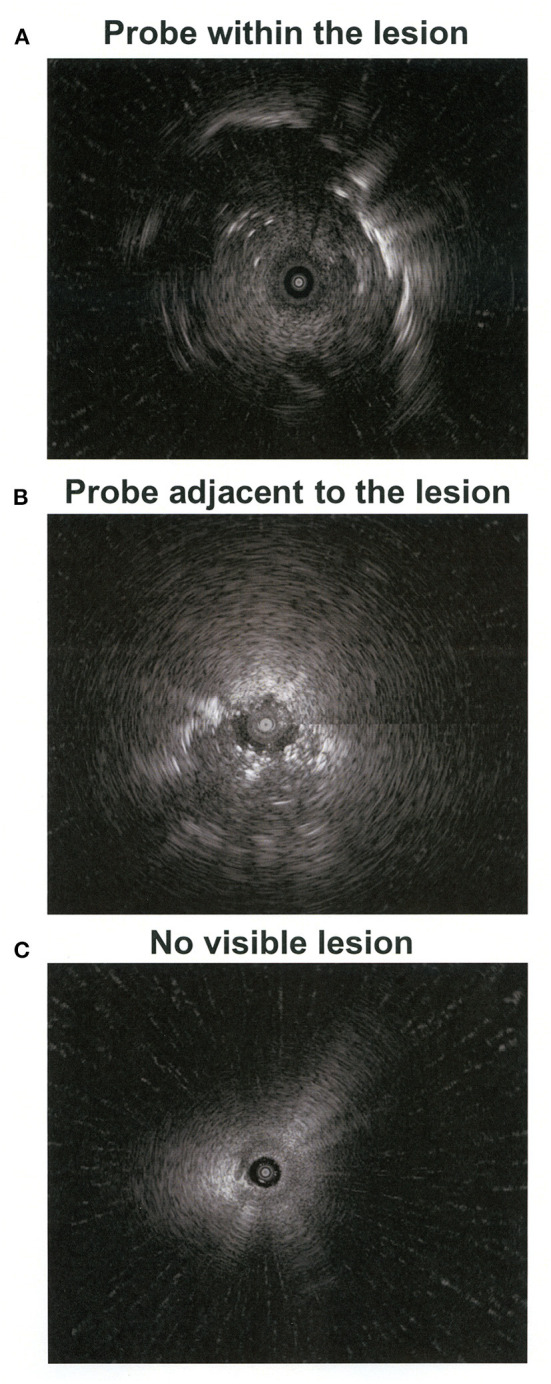
Images of the radial-probe endobronchial ultrasound. The central black cycles **(A**–**C)** indicate the probe position and the hyperechoic areas **(A,B)** show the lesion.

Bronchoscopy was performed under fluoroscopic guidance by a team of pulmonologists. The airway was visualized using the VBN system, and the thin bronchoscope was advanced as close as possible to the target lesion. The EBUS probe within a guide sheath was introduced via the bronchoscope's working channel, and then both were advanced as a single unit until the lesion was visualized under fluoroscopy as previously described (12). After confirming the EBUS view, the radial EBUS probe was removed, leaving the guide sheath in position. The radial EBUS views were categorized into three types of findings: (1) radial probe within the lesion, (2) radial probe adjacent to the lesion, or (3) no visible lesion (Figures 1A–C) (7).

We substituted the thin bronchoscope with the ultrathin instrument when the EBUS view showed that the radial probe's position was adjacent to the target lesion or when it showed no visible lesion. After removing the thin bronchoscope, we advanced the ultrathin bronchoscope up to the vicinity of the lesion guided by the VBN system and then inserted the radial EBUS probe through the bronchoscope channel and placed it close to the lesion. After confirming the radial EBUS view, we took five biopsy samples under fluoroscopy using forceps (FB-233D, Olympus, Tokyo, Japan) followed by brushing and washing with physiological saline.

### Criteria of Diagnostic Bronchoscopy

The bronchoscopy procedure was considered diagnostic in cases in which the pathologist reported the diagnosis of a definite disease and, in cases of organizing pneumonia in which the size of the biopsy tissue sample was appropriate, and the pulmonary lesion disappeared or reduced in size during follow-up. The procedure of bronchoscopy was considered non-diagnostic in cases of organizing pneumonia in which the EBUS probe was not within the target lesion, the size of the biopsy sample was not sufficiently large, and/or in cases with undefined diagnosis in which the lesion size remained unchanged during follow-up and specimens collected by CT-guided biopsy or surgery were not available.

### Statistical Analysis

The SPSS statistics software version 23 (SPSS Inc., Chicago, IL) was used in all statistical analyses. Chi-square was used to calculate differences between groups and the Mann-Whitney *U* test to compare continuous variables. Factors contributing to diagnosis were evaluated by logistic analysis. *P* < 0.05 was considered a significant difference.

## Results

**Figure 2:**
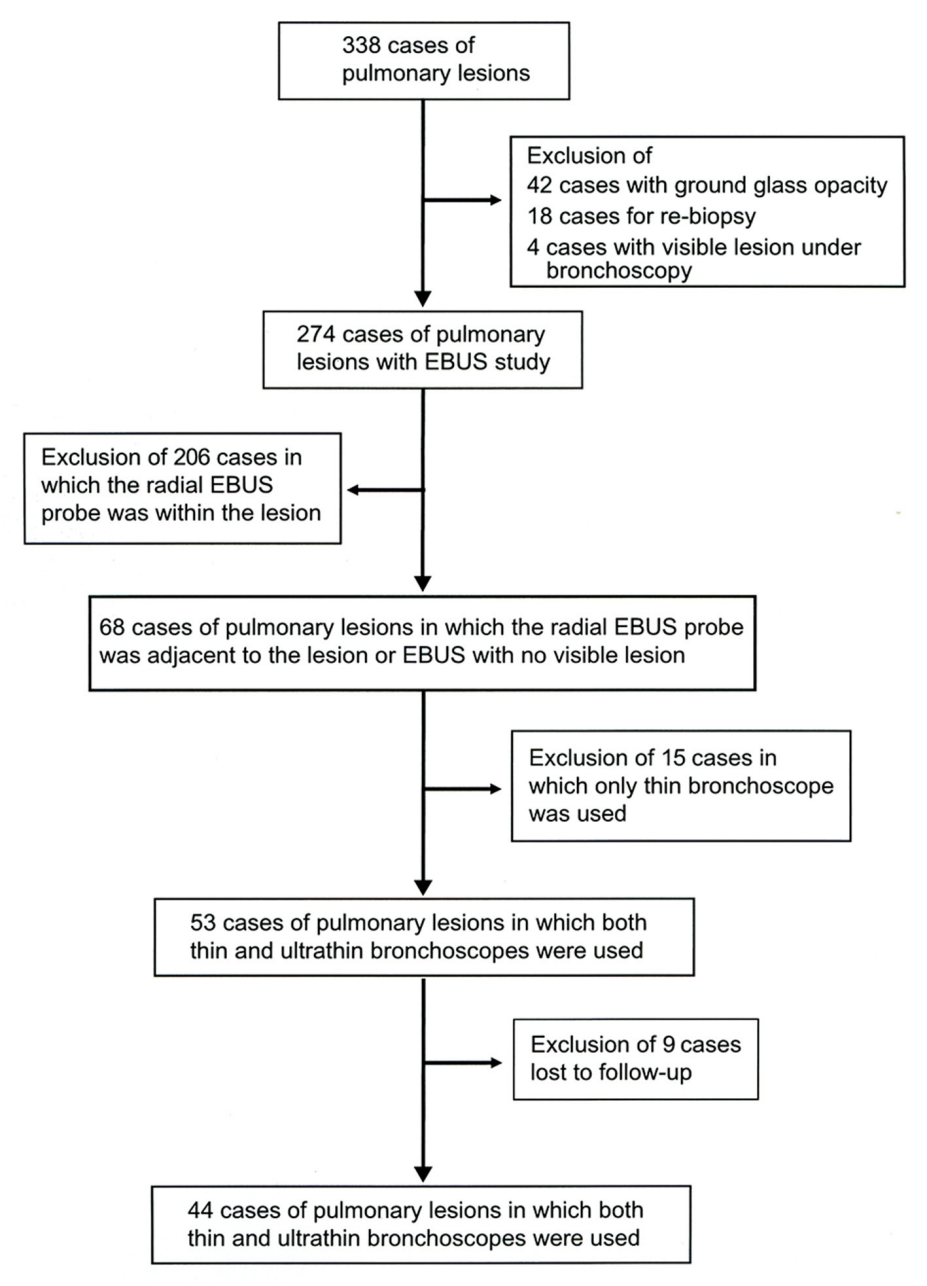
Study selection criteria. The records of 338 patients with pulmonary lesions that underwent multimodal bronchoscopy were assessed. The study included 44 cases in which the thin bronchoscope was substituted by the ultrathin bronchoscope on a need basis during the bronchoscopy procedure.

There were 338 patients with pulmonary lesions during the period of investigation. Sixty-four patients were excluded for the presence of ground-glass opacity on high-resolution CT because the examination purpose was re-biopsy or because the lesion was bronchoscopically visible (Figure 2). The remaining 274 patients underwent bronchoscopy using a thin bronchoscope and radial EBUS probe. Among these patients, the EBUS views showed the radial EBUS probe's position within the target lesion in 206, adjacent to the target lesion in 28, and showed no visible lesion in 40 cases. We substituted the thin bronchoscope in 16 of 28 cases in which the radial probe was adjacent to the lesion and in 37 of 40 cases in which the lesion was not visible. Although bronchoscope substitution was possible in 53 cases, subsequent analysis was possible only in 44 cases because nine were lost to follow-up (Figure 2). The pulmonary lesions' median diameter in all 44 subjects was 17.5 mm (6.0–52.0 mm), 28 of them having pulmonary lesions of <20 mm in diameter (Table 1). The lesions were peripheral in 37, showed CT bronchus-sign in 26 and cavitation in three cases.

**Table 1:** Characteristics of the patients.

**Variables**	**Age and No of cases**
No of patients	44
Median age (years, range)	74 (28–90)
Sex	
Male	29
Female	15
Lesion size (mm, median range)	
<10	7
10–20	21
>20	16
Radiological Location	
Central	1
Intermediate	6
Peripheral	37
Radiological findings	
Visible by plain radiography	28
Invisible by plain radiography	16
Nodules	44
With cavitation	3
With bronchus sign	26
Diagnosis	
= Lung malignant tumors	25
Lung primary cancer	
- adenocarcinoma	11
- squamous cell carcinoma	6
- large cell neuroendocrine carcinoma	2
- adeno-neuroendocrine carcinoma	1
- undefined histological type	1
Small cell lung cancer	3
Lung metastatic tumor	1
= Lung benign disease	14
Cryptococcus	4
Non-tuberculous Mycobacteriosis	1
Sarcoidosis	1
Organizing pneumonia	
With sufficient size of biopsy tissue	1
No sufficient size of biopsy tissue	7
= Undefined diagnosis	5

**Figure 3:**
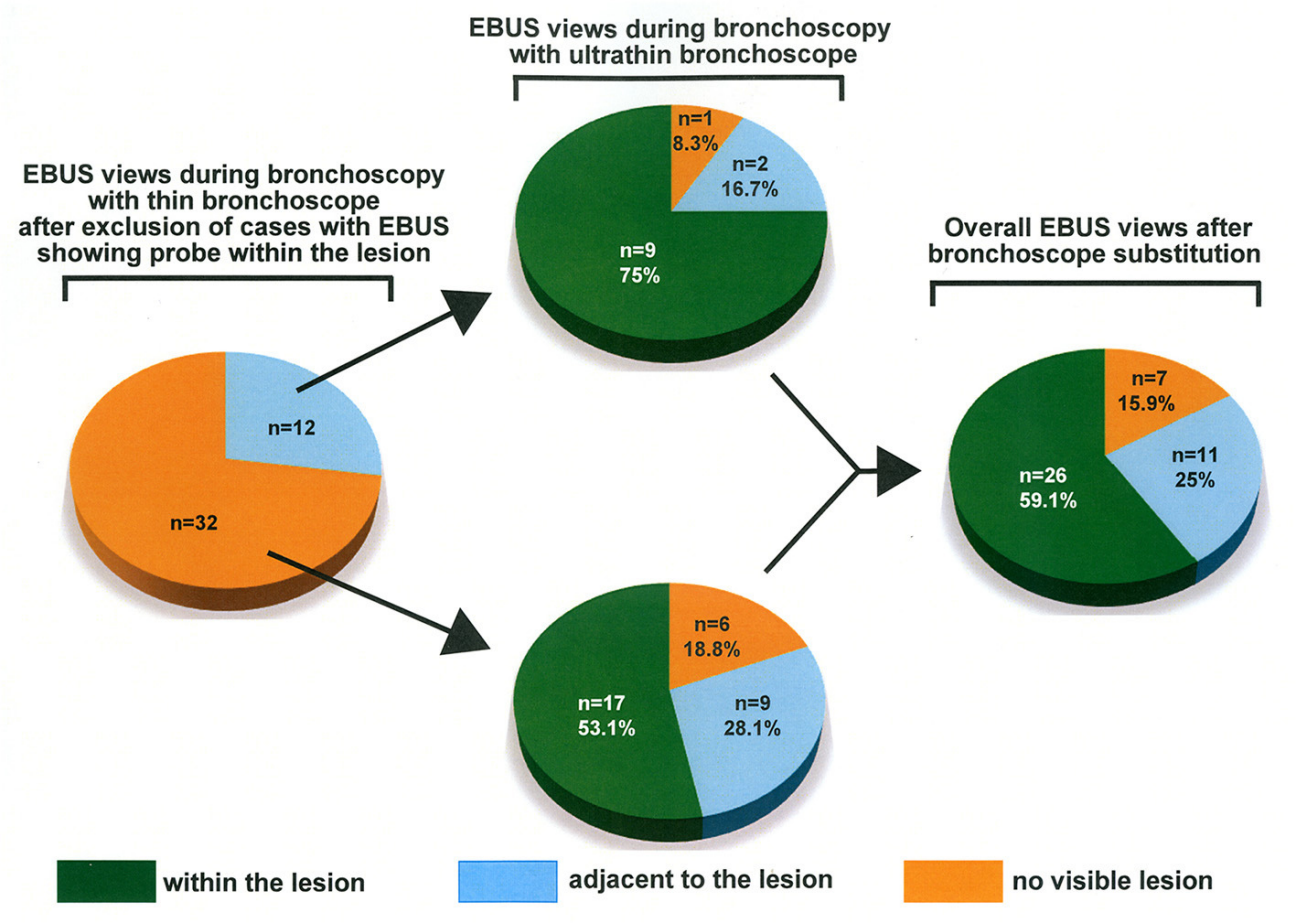
Changes in EBUS view after bronchoscope substitution. The thin bronchoscope was substituted by the ultrathin bronchoscope in 44 patients in whom the EBUS images showed the probe adjacent to the lesion or no visible lesion.

During the bronchoscopy procedure with a standard thin bronchoscope, the EBUS views showed that the radial probe's position was adjacent to the target lesion in 12 cases and no visible lesion in 32 cases. Among the 12 patients whose EBUS views showed the radial probe adjacent to the lesion during the procedure with a thin bronchoscope, the radial probe's position displayed by the EBUS view changed to within the target lesion in nine cases after using the ultrathin bronchoscope (Figure 3). Also, among the 32 patients whose EBUS views showed no visible lesion using a thin bronchoscope, the EBUS views showed the radial probe within the lesion in 17 cases or adjacent to the lesion in nine cases after substitution by ultrathin bronchoscope (Figure 3). In one case in which the EBUS showed the radial probe adjacent to the lesion using a thin bronchoscope, the EBUS views showed no visible lesion after device substitution by the ultrathin bronchoscope. This observation was due to bleeding from the lesion after the insertion of the radial-probe EBUS.

The diagnostic yield was 65.9% in all cases and 57.1% in cases with a pulmonary lesion of <20 mm in diameter (Table 2). The diagnostic yield was 80.8% when the EBUS view showed that the position of the radial probe was within the target lesion, 72.7% when the radial probe was located adjacent to the target lesion, and 0% when there were no visible lesions during bronchoscopy with ultrathin bronchoscope (Table 2). Twenty-five patients had confirmed diagnosis of malignancy, and 14 confirmed benign disease.

**Table 2:** Diagnostic yield by endo-bronchial ultrasound (EBUS) view and lesion size.

	**All cases**	**Cases with diagnostic bronchoscopy**	**Cases with non-diagnostic bronchoscopy**	**Overall Diagnostic yield (%)**
EBUS view				
Within the lesion	26	21	5	80.8
Adjacent to the lesion	11	8	3	72.7
No visible lesion	7	0	7	0.0
All views	44	29	15	65.9
Lesion size				
<10 mm	7	3	4	42.9
10–20 mm	21	13	8	61.9
>20 mm	16	13	3	81.3

The size of the pulmonary lesion, frequency at which the radial probe's position was within the target lesion during ultrathin bronchoscope, and presence of CT bronchus-sign on CT scan were significantly different between patients with and without a confirmed diagnosis by multimodal bronchoscopy (Table 3). However, the number of radiologically confirmed pulmonary lesions, and the number of lesions approachable by fluoroscopy were not significantly different between both groups (Table 3). Logistic analysis including lesion size, position of the radial probe within the target lesion, presence of CT bronchus-sign, and approachability by fluoroscopy showed that the position of the radial probe within the target lesion is the most significant contributing factor for diagnostic confirmation of lesions during multimodal bronchoscopy using an ultrathin bronchoscope (Table 4).

**Table 3:** Univariate analysis of determinant factors of diagnosis.

**Diagnostic factors**	**Diagnostic bronchoscopy**	**Non-diagnostic bronchoscopy**	***P*-values**
Lesion size	19.0	14.0	0.041
Within target lesion by EBUS through ultrathin bronchoscopy	72.4% (21/29)	33.3% (5/15)	0.012
Visible lesion on plain chest radiography	68.9% (20/29)	58.3% (8/15)	0.30
Approachability by fluoroscopy	68.9% (20/29)	40.0% (6/15)	0.064
Positivity of CT bronchus sign	72.4% (21/29)	40.0% (6/15)	0.036

**Table 4:** Multivariate analysis of determinant factors of diagnosis.

**Diagnostic factors**	**Adjusted odds ratio (95% confidence interval)**	***P*-values**
Lesion size	1.017 (0.905–1.142)	0.782
Within target lesion by EBUS through ultrathin bronchoscopy	0.268 (0.062–1.154)	0.077
Approachability by fluoroscopy	1.875 (0.395–8.870)	0.427
Positivity of CT bronchus-sign	0.462 (0.093–2.305)	0.347

## Discussion

Previous studies have shown that the radial EBUS probe's position during multimodal bronchoscopy using standard thin bronchoscopes predicts a high diagnostic yield of peripheral pulmonary lesions (4, 13). The diagnostic yield increases to more than 70% when the radial EBUS probe's position is within the pulmonary lesion, whereas it drops to <20% when the radial probe is located adjacent to or outside the lesion (4). These observations underscore the importance of placing the bronchoscope as close as possible to the radial probe within the target lesion.

Because there already exists evidence demonstrating the superiority of ultrathin bronchoscope over the thin device (10), we hypothesized that the thin device's substitution by the ultrathin bronchoscope during bronchoscopy procedures would further improve the radial probe positioning and consequently the diagnostic yield of pulmonary lesions. Before substituting the thin bronchoscope, the radial probe's position was adjacent to the lesion (27.3%) or outside the lesion (72.7%) in a high percentage of the patients. Although we performed no transbronchial lung biopsy at this time to avoid bleeding before using the ultrathin bronchoscope, there is a report showing a very low diagnostic yield (19.5%) in this population (4). Consistent with our hypothesis, we observed a significant improvement in the radial probe position with the lesion after substituting the thin device with the ultrathin bronchoscope. In addition, the overall diagnostic yield was higher than 80% in patients in whom the position of the radial EBUS probe was within the lesion and higher than 72% in cases in which the EBUS probe was adjacent to the lesion after bronchoscope substitution. Based on these results, we strongly recommend substituting the thin diagnostic device with the ultrathin bronchoscope when the EBUS shows no visible lesion.

An obvious advantage of the ultrathin bronchoscope is its major accessibility to the peripheral bronchial segments (14). Oki et al. reported that access to fourth-order bronchial segments is possible using the thin bronchoscope, whereas the ultrathin bronchoscope may allow access to up to fifth-order bronchial segments (14). In the present study, we used the recently developed MP290F-type ultrathin bronchoscope that has the working channel larger (1.7-mm vs. 1.2-mm) than the conventional XP260F-type ultrathin bronchoscope, and therefore, it enables the passage of EBUS-probe or 1.5-mm forceps to collect sufficient diagnostic materials. Although the risk of hemorrhage increases by performing biopsy with 1.5-mm forceps, the ultrathin bronchoscope itself can be used for hemostasis by applying firm pressure with its tip on the bronchial segment. With this maneuver, we had seen no case of severe hemorrhagic complications using large-sized forceps. Overall, these observations suggest that the superior performance of ultrathin bronchoscope compared to the thin bronchoscope during multimodal bronchoscopy procedures may be explained by its major accessibility to peripheral bronchial segments and its appropriate working channel size. It is worth noting that, compared to the standard thin bronchoscope, the ultrathin bronchoscope has several disadvantages, including the inability to use ancillary instruments (guide sheath, catheter for anesthesia instillation) and the limited intra-bronchial field of view (7). Therefore, we recommend using a standard thin bronchoscope and then using an ultrathin bronchoscope as a substitute on a need basis in cases where the radial EBUS probe's appropriate positioning is difficult. In addition, there are cases in which the diagnosis is difficult using the conventional thin bronchoscope despite the EBUS probe's location within the lesion (12, 15). The substitution of the thin bronchoscope by the ultrathin device may also be useful in these difficult cases.

## Limitations

The study's retrospective nature, focus on a single-institution population, lack of pathological evaluation before bronchoscope substitution, and the economic and time-consuming burden of bronchoscope substitution are the main limitations of the present study.

## Conclusions

The present study results showed for the first time that substitution of the standard thin bronchoscope by the ultrathin device on a need basis improves the position of the radial endobronchial ultrasound probe and the diagnostic yield of pulmonary lesions during multimodal diagnostic bronchoscopy.

## Data Availability Statement

The original contributions generated for this study are included in the article/supplementary material, further inquiries can be directed to the corresponding author/s.

## Ethics Statement

The studies involving human participants were reviewed and approved by The Ethics Committee for Clinical Investigation of Matsusaka Municipal Hospital approved the study protocol (Approval No J-58-191101-8-7) and the study was performed following the Principles of the Helsinki Declaration. The patients/participants provided their written informed consent to participate in this study. Written informed consent was obtained from the individual(s) for the publication of any potentially identifiable images or data included in this article.

## Author Contributions

OH, OT, FA, and YNi contributed to the conceptualization and idea of the study. KFuj, KI, TS, KFur, YNi, and YS contributed to the resources, supervision, and data analysis. YNi prepared the first draft of the manuscript. TK, EG, CD'A-G, TY, and FA made an intellectual contribution and edited the manuscript. OH and EG are the guarantors of the paper. All authors contributed to the article and approved the submitted version.

## Conflict of Interest

The authors declare that the research was conducted in the absence of any commercial or financial relationships that could be construed as a potential conflict of interest.
